# MicroRNAs Regulate Cellular ATP Levels by Targeting Mitochondrial Energy Metabolism Genes during C2C12 Myoblast Differentiation

**DOI:** 10.1371/journal.pone.0127850

**Published:** 2015-05-26

**Authors:** Puntita Siengdee, Nares Trakooljul, Eduard Murani, Manfred Schwerin, Klaus Wimmers, Siriluck Ponsuksili

**Affiliations:** 1 Research Institute for the Biology of Farm Animals (FBN), Research unit Functional Genomics, Dummerstorf, Germany; 2 Research Institute for the Biology of Farm Animals (FBN), Research Unit Molecular Biology, Dummerstorf, Germany; Huazhong Agricultural University, CHINA

## Abstract

In our previous study, we identified an miRNA regulatory network involved in energy metabolism in porcine muscle. To better understand the involvement of miRNAs in cellular ATP production and energy metabolism, here we used C2C12 myoblasts, in which ATP levels increase during differentiation, to identify miRNAs modulating these processes. ATP level, miRNA and mRNA microarray expression profiles during C2C12 differentiation into myotubes were assessed. The results suggest 14 miRNAs (miR-423-3p, miR-17, miR-130b, miR-301a/b, miR-345, miR-15a, miR-16a, miR-128, miR-615, miR-1968, miR-1a/b, and miR-194) as cellular ATP regulators targeting genes involved in mitochondrial energy metabolism (*Cox4i2*, *Cox6a2*, *Ndufb7*, *Ndufs4*, *Ndufs5*, and *Ndufv1*) during C2C12 differentiation. Among these, miR-423-3p showed a high inverse correlation with increasing ATP levels. Besides having implications in promoting cell growth and cell cycle progression, its function in cellular ATP regulation is yet unknown. Therefore, miR-423-3p was selected and validated for the function together with its potential target, Cox6a2. Overexpression of miR-423-3p in C2C12 myogenic differentiation lead to decreased cellular ATP level and decreased expression of Cox6a2 compared to the negative control. These results suggest miR-423-3p as a novel regulator of ATP/energy metabolism by targeting Cox6a2.

## Introduction

MicroRNAs (miRNAs) are evolutionarily conserved, short (~22 nucleotides), single-stranded, non-coding RNA molecules that regulate gene expression often by degrading or repressing translation of target mRNAs. miRNAs contain a 5′ “seed sequence” (nucleotide positions 2–8) used to predict binding sites on the 3′-UTR of target genes; however, the entire miRNA sequence can influence its binding affinity and effects. Functional miRNAs undergo biogenesis pathways including transcription, pre-processing, maturation, and, finally, formation of the RNA-induced silencing complex (RISC) [1–4]. Mature miRNAs play important roles in regulating of diverse biological processes including cellular development, differentiation, growth, proliferation, apoptosis, and metabolism [5–8]. Further, accumulating evidence indicates that miRNAs are involved in numerous regulatory networks. For example, estrogen-related receptor γ (ERRγ) establishes a nuclear receptor/miRNA regulatory network dictating the fiber-type composition in muscle [9]. The involvement of miRNAs in many cellular processes implies a likely contribution to energy metabolism. Energy metabolism produces ATP, the energy source vital to cellular functions, through several pathways including oxidative phosphorylation in the mitochondria. C2C12 cells have been widely used as a model to study myoblast differentiation and energy metabolic pathways. For C2C12 cell lines it was shown that some miRNAs are under control of transcriptional regulators of cellular energy metabolism; PPARβ/δ and ERRγ [9] or some metabolic transcript genes, YY1, eIF4E3, and PDCD10 [10]. Conversely, a number of miRNAs were demonstrated to control mitochondrial metabolism, through direct and indirect mechanisms [11–13]. MiR-20a and miR-106b were found to suppress the expression of the autophagy gene ULK1 which has key functions in mitochondrial respiration and against oxidative stress of the cells [11]. During myogenic differentiation of myoblast C2C12 cells miR-494 function on mitochondrial biogenesis by down-regulating mtTFA and Foxj3 [12].

We previously identified an miRNA-dependent regulatory network implicated in energy metabolism in porcine muscle [14]. However, knowledge on which miRNAs are involved in ATP production/energy metabolism remains scarce, and functional validation is needed to confirm the contribution of miRNAs to these processes.

Here, we used a myogenesis model to study miRNAs regulating cellular energy metabolism. Myogenesis is a multistep pathway in which myoblasts form from mesodermal precursor cells, withdraw from the cell cycle, express muscle-specific genes, and, eventually, differentiate into myotubes [15,16]. To identify miRNAs that can modulate expression of genes involved in energy metabolism, we performed correlation analyses between ATP levels and both miRNA and mRNA expression profiles during the C2C12 murine myotube differentiation. The resulting list of ATP/miRNA/mRNA correlation interactions revealed several candidate miRNAs, from which miR-423-3p was selected for functional validation.

## Materials and Methods

### Cell culture

**Fig 1:**
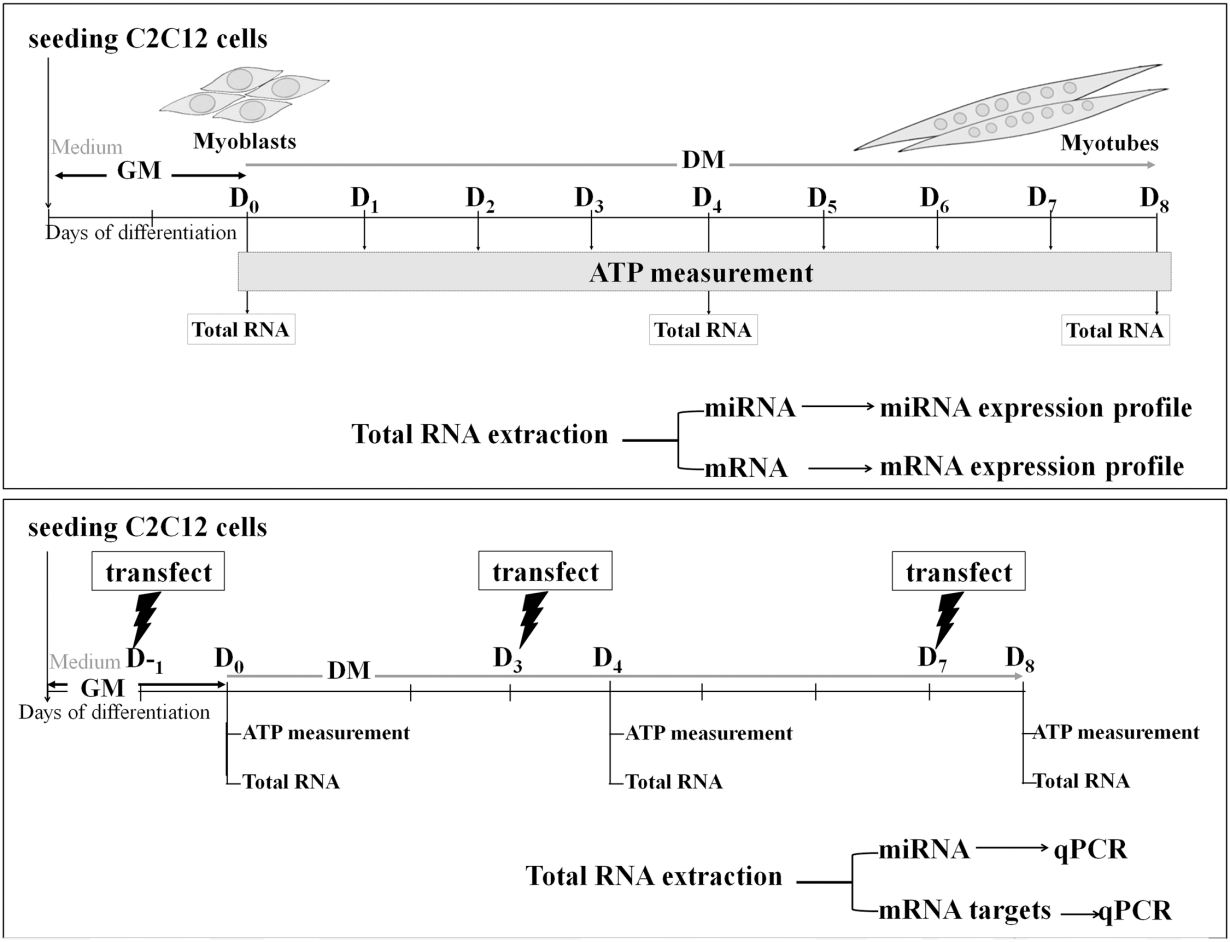
Experimental scheme. (A) ATP measurement and expression profiling of microRNA and target mRNA. C2C12 myoblasts were maintained in growth medium (GM) for 2 days (80–90% confluence). Myogenic differentiation was then induced from D0 to D8 by switching to differentiated medium (DM). Cells were harvested for (1) intracellular ATP measurement (2) expression profiling of miRNA (Affymetrix GeneChip miRNA 3.0 Array) and target mRNAs (Qiagen RT2 Profiler PCR Array focusing on mitochondrial energy metabolism genes). (B) miR-423-3p overexpression by transfection of synthetic miR-423-3p mimic. C2C12 myoblasts were transfected with miR-423-3p mimic 24 hours prior to myotube induction (D -1), D3 and D7 post-induction and were then collected on D0, D4, and D8, respectively, for miRNA and target gene qPCR as well as ATP measurement.

Mouse skeletal muscle C2C12 myoblasts passage no. 7–9 (ATCC CRL1772) were cultured in Dulbecco's modified Eagle's medium (DMEM, Invitrogen) containing 1 g/L glucose and supplemented with 10% FBS (PAA). At 80–90% confluence, myogenic differentiation was induced by switching to 2% (horse) serum-supplemented DMEM. Differentiation medium was changed every day during the course of myotube induction (see experimental scheme, Fig 1A). All cultures were maintained at 37°C under 5% CO_2_, and all media were supplemented with 1% penicillin/streptomycin (Biochrom).

### Bioluminescent luciferase assay

Intracellular ATP content was measured during the induction of myogenic differentiation on days 0 (D0) to 8 (D8) using an Adenosine 5'-triphosphate Bioluminescent Assay kit according to the manufacturer’s recommendations (Sigma). Briefly, C2C12 myoblasts were grown in 10 mL growth medium in a 75-cm^2^ flask at a density of 4×10^5^ cells/flask. Cultured cells were harvested at 24-hour intervals from D0 to D8 post-induction for an ATP measurement (Fig 1A). Cell suspensions were adjusted to a concentration of 5×10^5^ cells/mL by the Trypan Blue solution (Sigma) exclusion test and resuspended in a lysis buffer from the kit. Cell lysate was mixed with ATP assay solution, and bioluminescence was measured immediately with a DTX 880 Multimode Detector (Beckman Coulter). All measurements were performed in triplicate. Cellular ATP content was calculated from an ATP calibration curve generated from ATP standard solutions.

### RNA isolation

Total RNA was extracted from cultured C2C12 cells using Tri-Reagent (Sigma-Aldrich, Germany) followed by an on-column DNase treatment. For small RNA isolation and enrichment, an miReasy Mini kit and an RNeasy MinElute Cleanup kit (Qiagen, Germany) were used according to the manufacturer’s protocols. RNA quality and quantity were assessed with an Agilent 2100 Bioanalyzer (Agilent) using an Agilent RNA 6000 Nano kit (total RNA) and an Agilent small RNA kit (small RNA).

### MicroRNA-microarray analysis

MicroRNA expression profiling was performed using Affymetrix Gene Chip Micro 3.0 Array (Affymetrix, Inc, Santa Clara, CA, USA) containing 16,772 entries representing hairpin precursor (miRBase v17) (in total 19,724 probe sets for detection most of miRNA from 153 species), which provides >3-log dynamic range, with >95% reproducibility and 85% transcript detection at 1.0 amol, for a total RNA input of 130–500 ng. A total of 9 enriched small-RNA pools derived from D0, D4, and D8 post-induction of C2C12 myoblasts (three each) were used in the array hybridizations. Each RNA pool was generated from 5 individual RNA samples extracted from independent cultures. 200 ng of small RNA were used in sample preparation with a FlashTag Biotin RNA Labeling Kit for Affymetrix GeneChip miRNA arrays (Genisphere). The labeled RNA was then hybridized for 16 hours to an Affymetrix GeneChip miRNA array according to the manufacturer’s recommendations (Affymetrix), washed and stained in the Affymetrix Fluidics Station 450, and scanned on the Affymetrix G3000 GeneArray Scanner. The image files were analyzed using the Affymetrix software (Expression Console), Robust Multi-array Average (RMA) background correction, log-2 transformations and quantile normalization methods implemented in JMP Genomics 5.1 were performed for data pre-processing, normalization, and statistical analysis.

### RT^2^ Profiler PCR Array

Expression levels of 89 genes functionally associated with mitochondrial energy metabolism were determined using Mouse Mitochondrial Energy Metabolism RT^2^ Profiler PCR Array (Qiagen). The array is a pre-optimized qPCR panel of pathway-focused genes in a 96-well plate format including five standard housekeeping genes. Briefly, the real-time PCR was performed according to manufacturer’s recommendations using SYBR Green PCR Master Mix and 20 ng (total RNA) cDNA/reaction well on a LightCycler 480 (Roche). The thermal cycler program was 10 min at 95°C, followed by 45 cycles of 15 sec at 95°C and 1 min at 60°C. Dissociation curve analysis was performed immediately after the last PCR amplification cycle.

### Validation of microRNA-microarray

Expression differences for differentially-expressed miRNAs identified from microarray analysis (mmu-miR-423-3p, mmu-miR-128-3p, and mmu-miR-301a-3p) were validated by two-step real-time PCR. First, single-stranded cDNA was synthesized from total RNA using a Megaplex RT Primers kit, Rodent Pool Set v3.0 (Life Technologies) containing rodent-specific stem-loop primers of 641 and 373 unique microRNAs for mouse and rat, respectively (see S1 Table for additional primer information). Real-time PCR was performed using a standard LightCycler 480 SYBR Green I Master (Roche) on the LightCycler 480 system (Roche). U6 was used as an internal standard and the relative abundance of miRNAs was calculated using a comparative threshold cycle ΔΔCt method [17]. Validation of mmu-miR-423-3p mimic transfection was carried out using the same method.

### mmu-miR-423-3p mimic transfection

Cells were seeded at a density of 1.5×105 cells/well on a 6-well plate in 2 mL of the growth medium. The C2C12 myoblasts were transfected with 150 nM mmu-miR-423-3p miScript miRNA mimic (5'-AGCUCGGUCUGAGGCCCCUCAGU -3', Qiagen) as double-stranded RNA oligonucleotides using the HiPerFect transfection reagent (Qiagen) at three different time points: 1 day prior to myotube induction and D3 and D7 post-induction (Fig 1B). Transfected myoblasts were harvested 24 hours post-transfection on D0, D4, and D8 post-induction for RNA extraction and qPCR. A random-sequence double-stranded RNA oligonucleotide was used as a negative control in all transfections (Qiagen). Transfection conditions were pre-optimized using the siGLO Green transfection indicator (Thermo Scientific) according to the manufacture’s recommendations.

### Bioinformatic and statistical analysis

We predicted targets using the computational software RNAhybrid (http://bibiserv.techfak.uni-bielefeld.de/rnahybrid), which detects the most energetically favorable hybridization sites of a small RNA (miRNA) within a large RNA (mRNA) [18,19]. Here, we tested the miRNA probe sets with the following parameters: energy cutoff (mfe) = -20 kcal/mol, allowing the G:U wobble base-pair, 1 mismatch seed, and ≤5 internal bulging nucleotides in the seed region. Identified targets were located on the 3′-UTR of genes. Statistical analysis for microRNA microarray and RT^2^ PCR array data was performed using a general linear model on JMP Genomics 6 (SAS Institute Inc., http://www.jmp.com). Adjustment for multiple tests across the Type 3 tests for the fixed effects was calculated using the post-hoc Tukey-Kramer test. For controlling false discovery rate, we chose the FDR according to Benjamini and Hochberg, 1995 [20]. Correlation coefficient analysis (r) between miRNA or mitochondria gene expression and ATP level was performed using SAS version 9.3. The expression data are available in the Gene Expression Omnibus public repository with the GEO accession number GSE52410: GSM1265694- GSM1265710

### Luciferase reporter assay

A 256 bp fragment encompassing two miR-423-3p binding sites was amplified from genomic DNA using XhoI-integrated forward primer, 5′-gcactcgagaccggttatgagcacccttg-3′ and NotI-reverse primer, 5′-taagcggccgctgcaggtggaaacatcacat-3′ designed from NM_009943. The fragment was cloned into the multiple cloning region at downstream of the Renilla luciferase reporter gene using the psiCHECKTM-2 vector system (Promega). The construct was validated by Sanger sequencing. For luciferase assay, 100 ng of DNA construct and 50 nM of miR-423-3p mimic were co-transfected into 2 x 10^4^ COS-7 cells seeded in a 96-well plate one-day prior to transfection using Attractene Transfection Reagent (Qiagen) according to the manufacturer’s recommendation. Firefly and *Renilla* activities were determined 48 h post transfection using the Dual-Glo Luciferase Assay System (Promega) and a DTX 880 Multimode Detector (Beckman Coulter, Germany). AllStars Negative control (Qiagen) was used in pairwise co-transfection as a negative control and normalization for the effects of endogenous miRNAs.

### siRNA transfection of *Cox6a2*

Synthetic siRNAs were pre-designed by Qiagen. A total of 4 pre-designed siRNAs (Qiagen) complementary to *Cox6a2* were tested first and the most effective siRNA was used (Mm_Cox6a2_5). The average values of negative `non-silencing control siRNA´ (AllStars Negative Control siRNA, Qiagen), `mock´, and `untreated´ were used as control. The experiments were set up in triplicate. Transfection of siRNA was carried out using the HiPerFect transfection reagent (Qiagen) at 150 nM final concentration at three different time points: 1 day prior to myotube induction and D3 and D7 post-induction. Forty-eight hours after siRNA transfection, cells were rinsed 2 times with PBS. One set of the transfected cells was harvested for monitoring the effect of gene silencing. Another set was used for ATP level and ADP/ATP ratio determination. We obtained the level of knockdown of cDNA using quantitative PCR (qPCR) (Roche, Germany) and normalized data using *Hprt1* and *Ppia* as internal controls. Therefore, the geometric means of raw threshold cycle (CT) values of these two genes were used for further calculations. All statistical analyses were performed using two-tailed Student's t-tests.

### ADP/ATP Ratio Bioluminescent Assay

After knockdown of *Cox6a2*, in addition of ATP measurement, the ADP/ATP ratio was also determined by bioluminescent, which can be used as an indicator of cell viability, necrosis and apoptosis. ADP/ATP ration was measured during the induction of myogenic differentiation on D0, D4 and D8 using ADP/ATP Ratio Assay Kit according to the manufacturer’s recommendations (Abcam). Briefly, C2C12 myoblasts were grown in 10 mL growth medium in a 75-cm2 flask at a density of 4×10^5^ cells/flask. Cultured cells were harvested at 24-hour intervals from D0, D4 and D8 post-induction of siRNA and controls. The nucleotides were released from cell suspensions by adding nucleotide releasing reagent. The ATP levels were measured. After 2 minutes the ADP in the wells was converted to ATP by adding of ADP converted reagent. The ration of ADP/ATP was calculated.

## Results

### Increased intracellular ATP during C2C12 myotube induction

**Fig 2:**
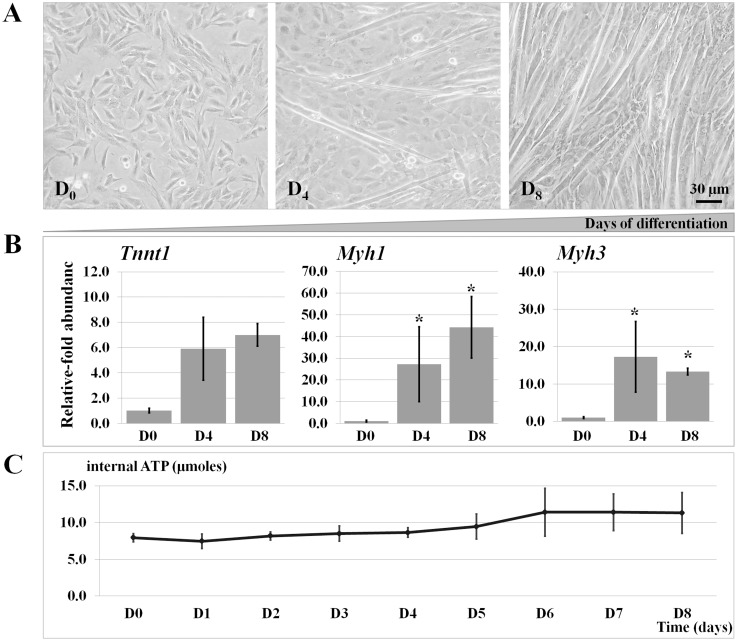
Characteristics of myogenic differentiation. (A) Morphological change of C2C12 myoblasts differentiating into myotubes at the post-induction D0 (undifferentiated mononucleated cells), D4 (elongated confluent cells), and D8 (long multinucleated myotubes). (B) Myogenic differentiation was accompanied by the up-regulation of Tnnt1, Myh1, and Myh3. (C) An increase in intracellular ATP level (μmoles) during the course of myogenic differentiation. The ATP level is shown as mean ± SEM (n = 5).

To identify a critical time point for a functional study of ATP-miRNA regulatory pathways, intracellular ATP content was measured during the myotube induction of C2C12 myoblasts using a luciferase assay. At D0, cells were mononucleated and 80–90% confluent. After 4 days post-induction in low serum medium, they gradually differentiated and, by D8, were predominantly multinucleated myotubes (Fig 2A). Induction of differentiation was confirmed by measuring mRNA expression of myogenic markers *Tnnt1*, *Myh1*, and *Myh3*, all of which were up-regulated (Fig 2B). Intracellular ATP content was determined in the differentiating cells at an interval of 24 hours during the course of induced differentiation (D0 to D8). ATP level increased gradually from D0–D5, increased sharply from D5–D6, and reached an approximate two-fold increase at D6–D8 compared to D0 (Fig 2C). ATP levels are significantly different between day 0 and day 8 (p = 0.014) as well as between day 4 and day 8 (p = 0.029), whereas the increment of ATP levels between day 0 and day 4 was subtle and did not reach the significant threshold.

### Altered miRNA expression during C2C12 myogenic differentiation

**Fig 3:**
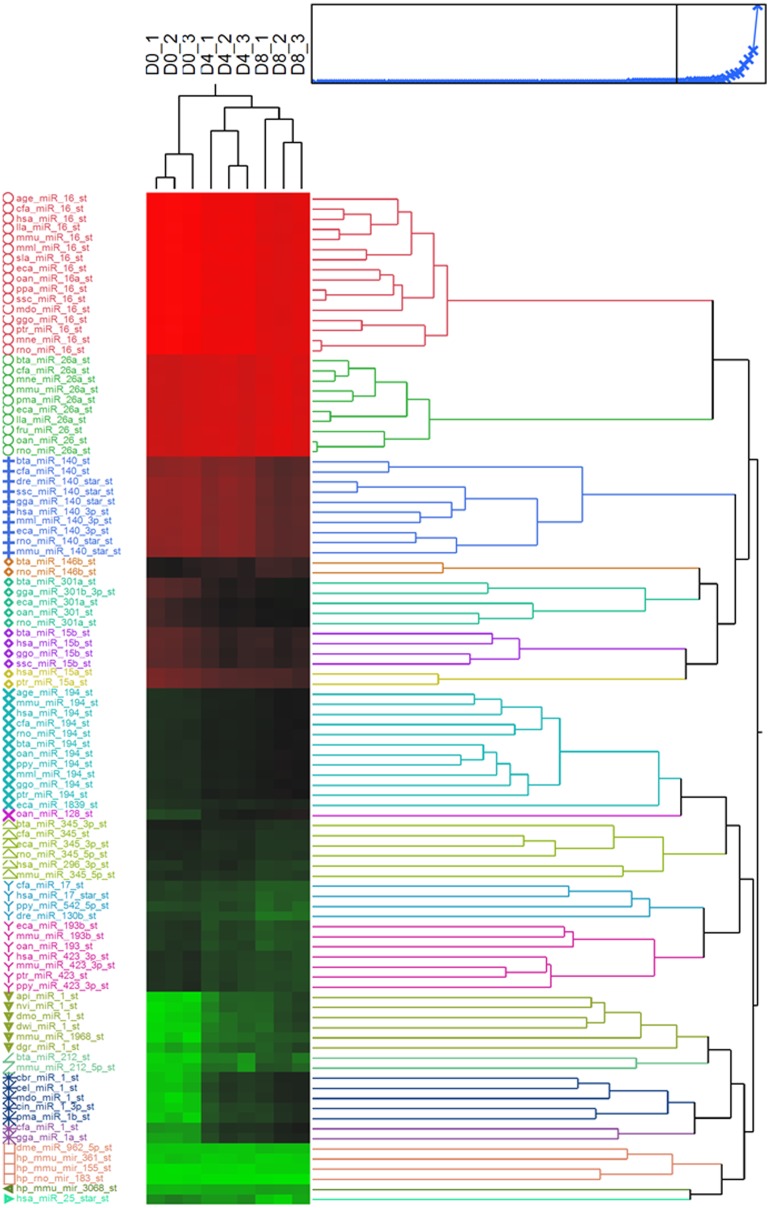
Hierarchical clustering of the top 100 miRNA probe sets which were differentially expressed at different time point of D0, D4, and D8 of C2C12 myoblasts cell.

**Fig 4:**
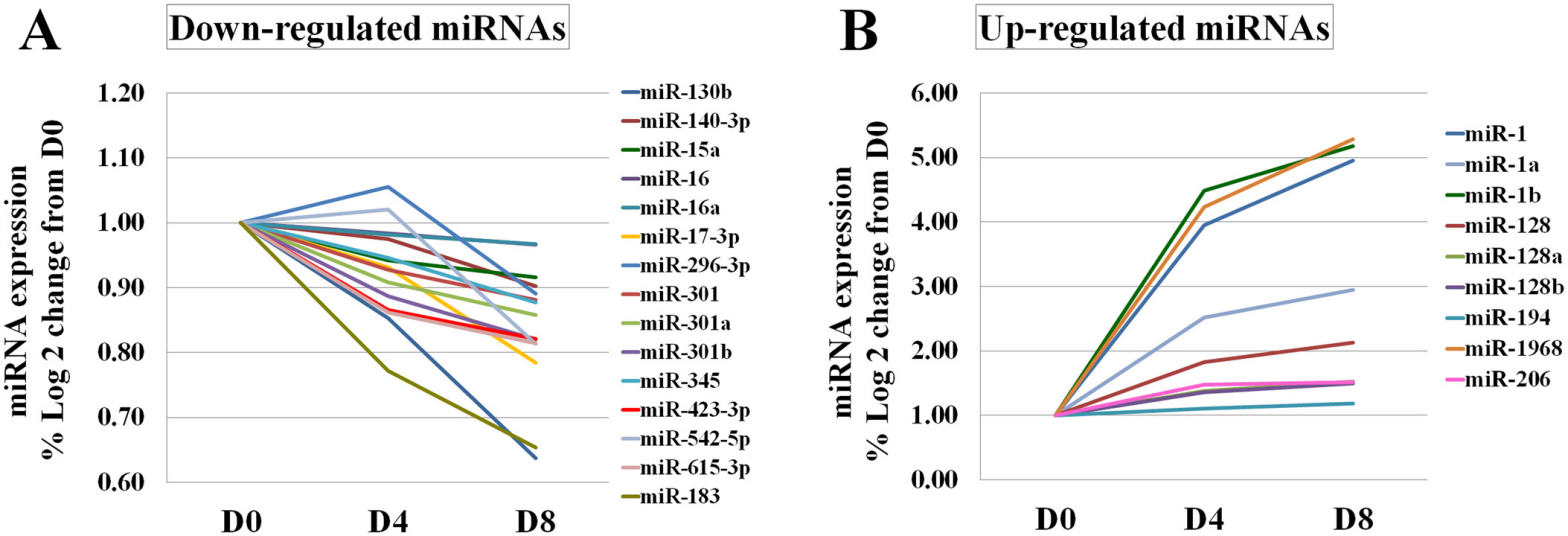
Alteration of miRNA expression in C2C12 myoblasts post-myotube induction. (A) 15 down-regulated miRNAs that were negatively correlated with the ATP level (correlation coefficient, r = -0.67 to -0.83, P< 0.05). (B) 9 up-regulated miRNAs that were positively correlated with the ATP level (r = 0.67 to 0.79, P< 0.05).

**Fig 5:**
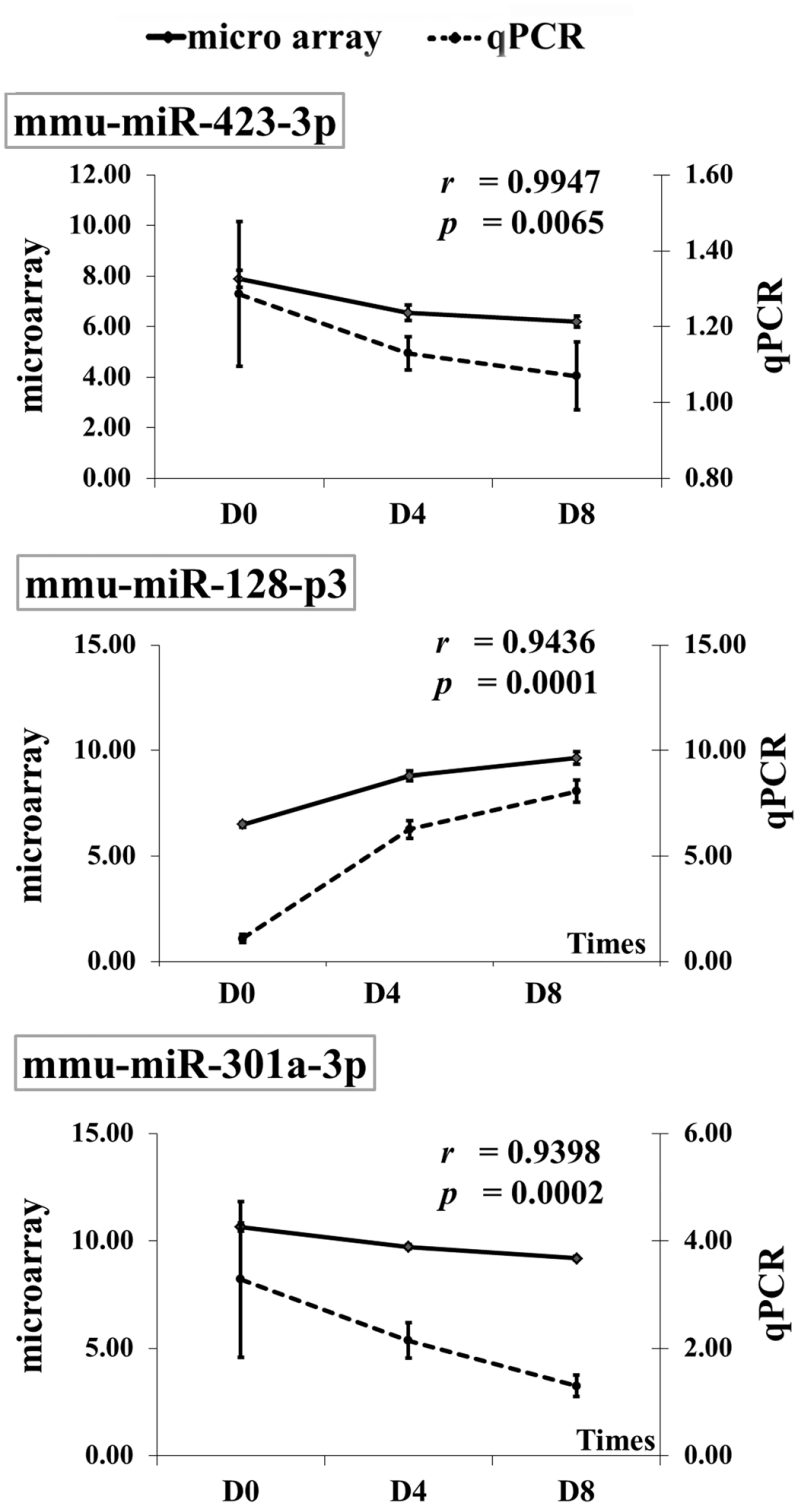
Confirmation of microRNA-microarray results by qPCR. miR-423-3p, miR-128-3p, and miR-301a-3p were selected for qPCR validation. Mean ± SEM (n = 4) of the log2 transformed microarray result (–•– on primary y-axis) and relative expression (2-ΔΔCt) derived from qPCR (—•—secondary y-axis) are overlaid. A correlation coefficient (r) and p-value are indicated.

Expression of miRNA during myogenic differentiation of C2C12 myoblasts was profiled at D0, D4, and D8 post-induction. After data pre-processing, 5,040 probes passed the quality control and filtering criteria and were analyzed further. To identify differentially-expressed miRNAs, the expression level of miRNAs was compared between pre- (D0) and post- (D4, D8) myotube induction. The hierarchical clustering of top 100 differentially-expressed miRNAs probe sets at different time points was shown (Fig 3). At D4 and D8, 179 and 188 probes, respectively, had significant expression changes from D0 (FDR ≤ 0.05). To focus on miRNA-ATP regulatory pathways, a correlation analysis between the expression of miRNA probes which significantly changes from D0 and ATP level was applied; this filtering resulted in 95 (D4) and 103 (D8) probes of interest. Of these, 46 probes representing 13 unique miRNAs (miR-301, miR-301a/b, miR-423-3p, miR-615-3p, miR-130b, miR-140-3p, miR-17-3p, miR-183, miR-345, miR-15a, miR-16, and miR-16a) were down-regulated (compared to D0) and negatively correlated with ATP at D4 post-induction. In contrast, 49 probes representing 10 miRNAs (miR-296, miR-128, miR-128a/b, miR-1968, miR-206, miR-194, miR-1968 and miR-1a/b) were up-regulated and positively correlated with ATP level at D4. At D8 post-induction, expression of 55 out of 103 probes was lower compared to D0. These corresponded to 15 unique down-regulated miRNAs whose expression level negatively correlated with intracellular ATP content (miR-130b, miR-140, miR-15a, miR-16, miR-16a, miR-17-3p, miR-296-3p, miR-301, miR-301a/b, miR-345-3p, miR-423-3p, miR-542, miR-615-3p, and miR-183). Expression of 48 probes was higher, corresponding to 9 up-regulated miRNAs positively correlated with the ATP level (miR-194, miR-1968, miR-206, miR-128, miR-128a/b and miR-1, miR-1a/b). Notably, among them, well-known muscle miRNAs miR-1 and miR-128 were highly up-regulated (>10-fold). The majority of differentially-expressed miRNAs were found at both D4 and D8 (Fig 4). miR-423-3p, miR-128-3p and miR-301a-3p were random selected for validation by real-time PCR. A highly correlation coefficient (0.93–0.99) between microarray and real-time PCR results was found (Fig 5).

### Identification of genes associated with cellular ATP content during C2C12 differentiation

To functionally link miRNAs to cellular ATP production, mRNA targets must be identified. We therefore performed an expression analysis of target genes on the same samples used in miRNA profiling. Here, the RT^2^ Profiler PCR Array system containing a panel of 89 mitochondrial energy metabolism-related genes was used. Analysis of this array identified 35 and 49 genes that were differentially expressed at D4 and D8, respectively, compared to D0. Further, a correlation analysis of these differentially-expressed genes with ATP content, performed as with the miRNA analysis, narrowed down the gene list to 13 genes significantly positively correlated with ATP level (r = 0.63–0.92, *p*-value < 0.05) (Table 1). Of these, the mRNA abundance of 7 out of 13 genes significantly increased as early as D4 and all 13 genes were ultimately up-regulated at D8. Among them 8 genes showed > 2-fold change at D4 and/or D8 post-myotube induction.

**Table 1:** List of differentially-expressed genes affecting ATP level during myogenic differentiation in C2C12 cells.

Gene symbol	Ref. sequence	Description	ATP correlation coefficient	P-value of correlation coefficient	Fold-change (D0)-(D4)	p-value (D0)-(D4)	FDR (D0)-(D4)	Fold-change (D0)-(D8)	p-value (D0)-(D8)	FDR (D0)-(D8)
***Cox4i2***	**NM_053091**	Cytochrome c oxidase subunit IV isoform 2	0.9234	0.0004	1.7275	0.0297	0.0890	2.2130	0.0050	0.0350
***Ndufs7***	**NM_029272**	NADH dehydrogenase (ubiquinone) Fe-S protein 7	0.8551	0.0033	1.2272	0.0644	0.1504	1.3591	0.0150	0.0626
***Ndufb7***	**NM_025843**	NADH dehydrogenase (ubiquinone) 1 beta subcomplex, 7	0.8314	0.0055	1.4096	0.0033	0.0279	1.6771	0.0005	0.0104
***Cox6a2***	**NM_009943**	Cytochrome c oxidase, subunit VI a, polypeptide 2	0.7727	0.0147	37.5828	0.0011	0.0142	57.3876	0.0002	0.0060
***Ndufs4***	**NM_010887**	NADH dehydrogenase (ubiquinone) Fe-S protein 4	0.7673	0.0158	1.7275	0.0257	0.0860	2.0411	0.0092	0.0478
***Ndufb3***	**NM_025597**	NADH dehydrogenase (ubiquinone) 1 beta subcomplex 3	0.7624	0.0169	1.1345	0.2691	0.4037	1.3560	0.0288	0.0873
***Ndufv1***	**NM_133666**	NADH dehydrogenase (ubiquinone) flavoprotein 1	0.7415	0.0222	1.5569	0.0052	0.0350	1.6888	0.0024	0.0217
***Bcs1l***	**NM_025784**	BCS1-like (yeast)	0.7417	0.0222	1.1163	0.0237	0.0828	1.1914	0.0047	0.0343
***Lhpp***	**NM_029609**	Phospholysine phosphohistidine inorganic pyrophosphate phosphatase	0.7161	0.0300	1.1991	0.2450	0.3874	1.5080	0.0265	0.0864
***Ndufs3***	**NM_026688**	NADH dehydrogenase (ubiquinone) Fe-S protein 3	0.7122	0.0313	1.3031	0.0526	0.1360	1.3941	0.0261	0.0862
***Ndufs5***	**NM_001030274**	NADH dehydrogenase (ubiquinone) Fe-S protein 5	0.7089	0.0325	1.4526	0.0051	0.0350	1.5977	0.0018	0.0180
***Ndufa2***	**NM_010885**	NADH dehydrogenase (ubiquinone) 1 alpha subcomplex, 2	0.7021	0.0350	1.1060	0.1315	0.2486	1.2449	0.0114	0.0538
***Atp6v0d2***	**NM_175406**	ATPase, H+ transporting, lysosomal V0 subunit D2	0.6921	0.0388	3.6774	0.2224	0.3647	18.2860	0.0059	0.0383

Data are shown as fold regulation levels compared to control group (D0) and normalized to mean of housekeeping genes (*Actb*, *B2m*, *Gapdh*, *Gusb*, and *Hsp90ab1*), then assessed for correlation with ATP levels.

### Associations between miRNAs and mitochondrial energy-metabolism genes

**Fig 6:**
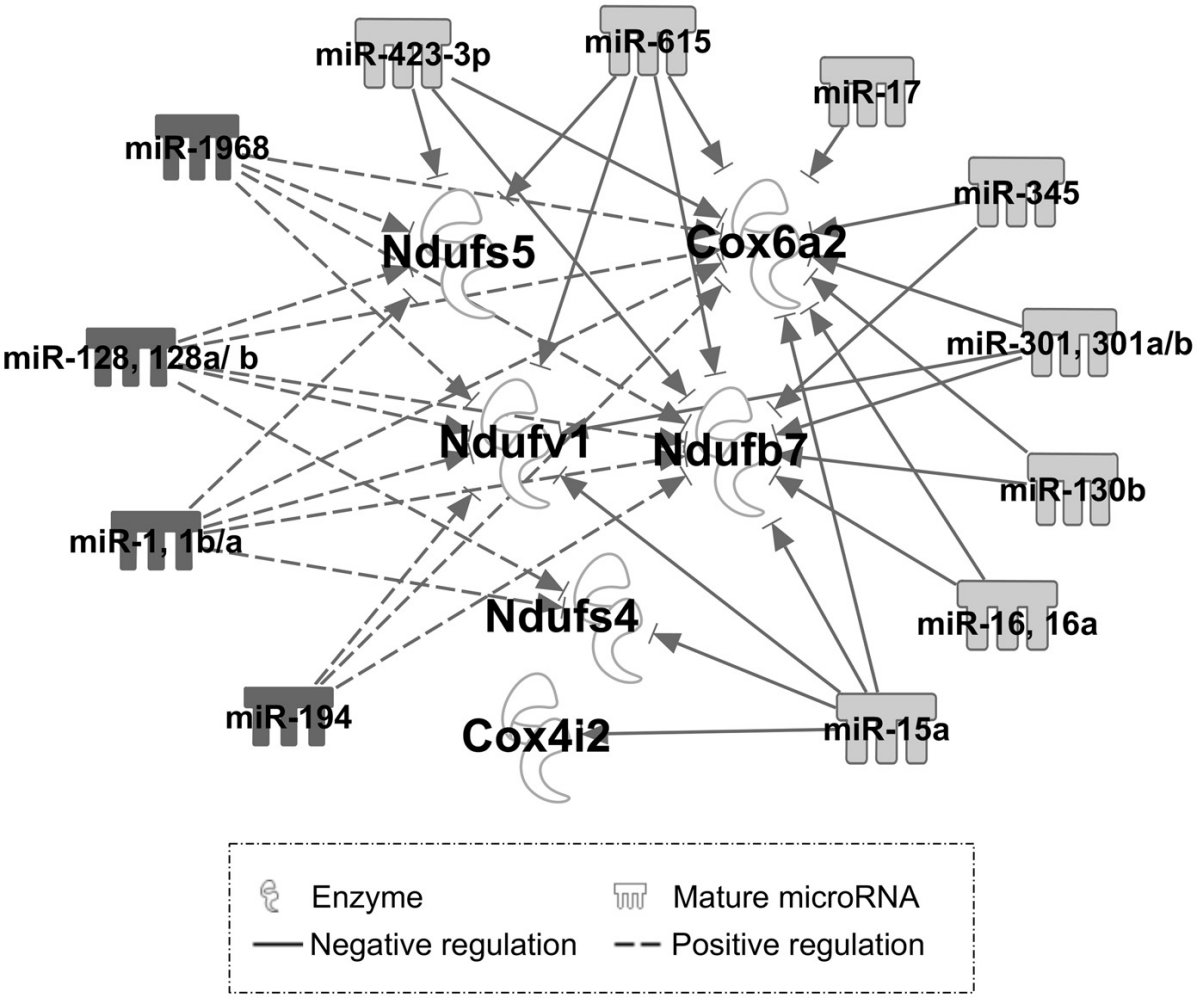
A regulatory network of differentially-expressed miRNAs and genes in C2C12 myogenesis, focusing on mitochondrial energy-metabolism pathways. Pearson correlation coefficient analysis identified a number of miRNAs-mRNA correlation interactions. Only those interactions in which both miRNAs and target mRNAs significantly associated with differentiation and ATP level (FDR < 0.1) during C2C12 myotube induction were utilized to model an miRNA-regulatory network (IPA pathway designer tool). White nodes indicate mRNA target; edges indicate regulatory interaction between miRNA and target gene. Solid line label denotes negative regulation with negative correlation interaction; dashed line denotes indirect positive regulation with positive correlation interaction.

A pairwise correlation analysis was carried out between the expression level of miRNAs (5,040 filtered probes) and 89 focused genes derived from the RT^2^ PCR array analysis. A Pearson correlation coefficient analysis revealed 4,260 significant correlation interactions (FDR < 0.05). Further filtering criteria were applied to consider only those interactions in which both miRNAs and target mRNAs were significantly associated with differentiation and ATP level (FDR < 0.1) of C2C12 myotube induction. These resulted in 168 positive and 77 negative correlation interactions. Among these, 14 miRNAs (miR-423-3p, miR-17, miR-130b, miR-301a/b, miR-345, miR-15a, miR-16a, miR-128, miR-615, miR-1968, miR-1a/b, and miR-194) and 6 target mRNAs (*Cox4i2*, *Cox6a2*, *Ndufb7*, *Ndufs4*, *Ndufs5*, and *Ndufv1*) were identified. The miRNAs and mRNAs with high correlation interactions included miR-1 and *Ndufv1* (r = 0.988, p = 5.486E-07) and miR-423-3p and *Cox6a2* (r = -0.971; p = 1.401E-05). Although most miRNAs inhibit their target mRNAs, some, particularly those miRNAs encoded within mRNAs, are positively correlated with expression of their targets [21]. However, positive correlations could also indicate a connection via as-yet-undetermined indirect regulations. Therefore, we included both positive and negative correlation interactions in an IPA pathway analysis (Fig 6). Additionally, a computational bioinformatic web tool (RNAhybrid) [18,19] was used to scan significant correlation pairs of miRNA-mRNA to obtain additional evidence for their functional links (Table 2). This analysis identified a miR-423-3p as an miRNA of interest for potential interactions with several mRNAs in energy metabolism.

**Table 2:** Predicted binding sites for miRNAs in the 3′-UTRs of their correlated mRNAs (RNAhybrid).

Regulatory interaction	Name	Sequence	Cox6a2	Ndufb7	Ndufs5	Ndufv1	Ndufs4	Cox4i2
negative	miR-423-3p	AGCUCGGUCUGAGGCCCCUCAGU	3 (-33.1 to -22.7)	1 (-20.2)	1 (-22.8)		4*	
	miR-17-3p	ACUGCAGUGAGGGCACUUGUAG	1 (-22.7)				4*	1*
	miR-301a-3p	CAGUGCAAUAGUAUUGUCAAAGC	n/a	n/a		n/a	1*	
	miR-345-3p	CCUGAACUAGGGGUCUGGAG	2 (-26.1 to -22.1)	1 (-20.9)		n/a		
	miR-345-3p	CCUGAACUAGGGGUCUGGAGAC	2 (-27.2 to -25.4)	1 (-20.9)		1*	4*	
	miR-15a-5p	UAGCAGCACAUAAUGGUUUGUG	1 (-18.7)	n/a		1 (-18.3)	3 (-20.8 to -18.7)	1 (-19.4)
	miR-16-5p	UAGCAGCACGUAAAUAUUGGCG	n/a	n/a			2*	
	miR-615-3p	UCCGAGCCUGGGUCUCCCUCUU	3 (-28.0 to -19.8)	1 (-19.1)	n/a	n/a	1*	
	miR-130b	CAGUGCAAUAAUGAAAGGGCAU	1 (-19.2)	n/a	2*		1*	
positive	miR-128-3p	UCACAGUGAACCGGUCUCUUU	1 (-22.6)	n/a	1 (-22.6)	n/a	1 (-20.6)	
	miR-1b	UGGAAUGUAAAGAAGUAUGGGU	n/a	n/a	n/a	n/a	5 (-23.1 to -18.0)	
	miR-1a	UGGAAUGUAAAGAAGUAUGUA	n/a	n/a	n/a	n/a	1 (-19.1)	
	miR-1-3p	UGGAAUGUAAAGAAGUAUGGAG	n/a	n/a	n/a	n/a	2 (-20.5 to -18.1)	
	miR-194-5p	UGUAACAGCAACUCCAUGUGGA	n/a	n/a	1*	n/a	4*	
	miR-1968-5p	UGCAGCUGUUAAGGAUGGUGGACU	2 (-23.5 to -20.0)	n/a	1 (-21.2)	1 (-22.7)	4*	2*

The number of binding sites at the 3´-UTR of significant correlated mRNAs is presented with the free energy hybridization of miRNA and target in parenthesis. n/a denotes no binding site available (predicted) at the 3´-UTR.

* denotes that binding sites were predicted while the negative correlation between miRNA and mRNA was not significant.

### miR-423-3p as an ATP-regulating miRNA candidate

**Fig 7:**
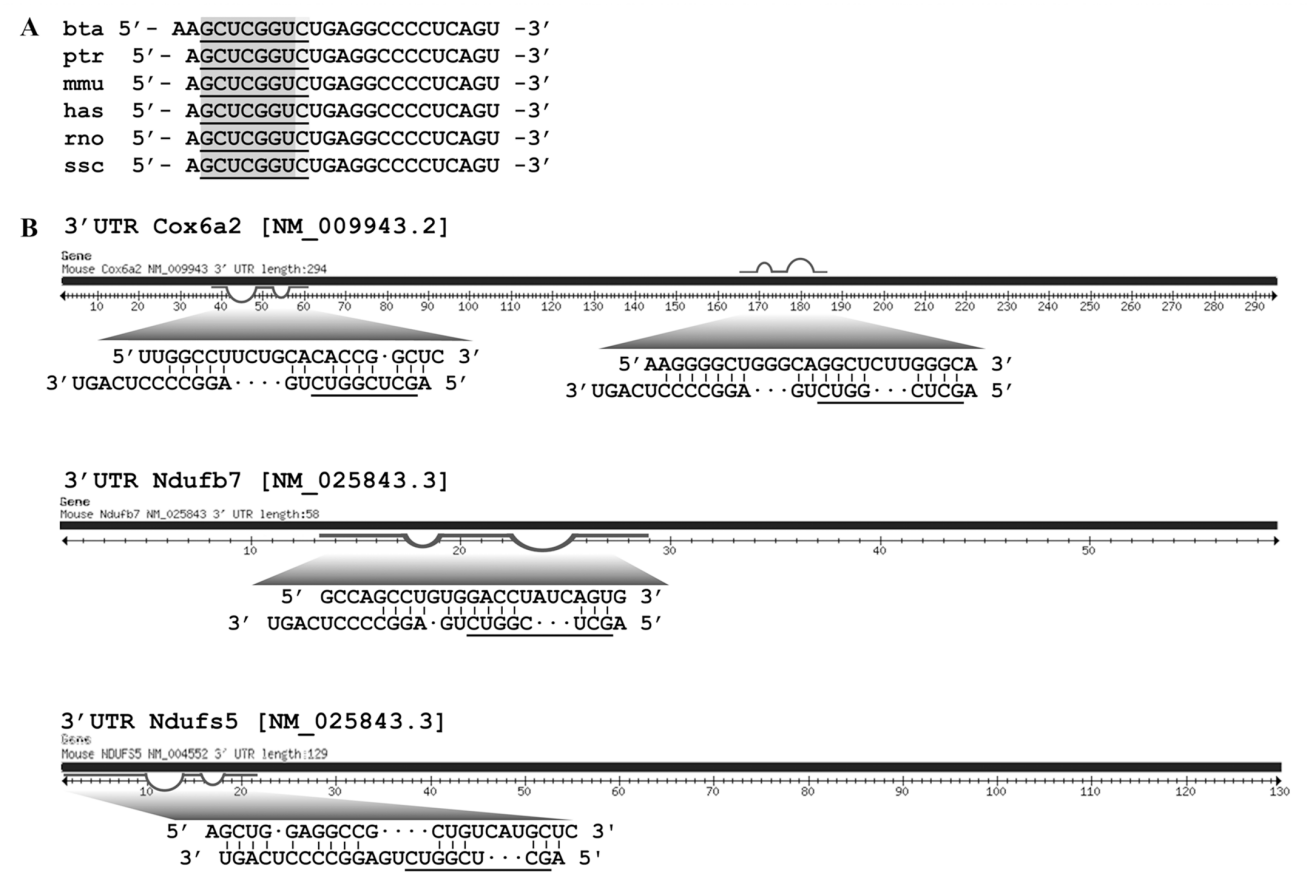
miR-423-3p as a candidate miRNA for functional validation. (A) Conservation of miRNA-423-3p across species; Bos taurus (bta), Pan troglodytes (ptr), Mus musculus (mmu), Homo sapiens (hsa), Rattus norvegicus (rno), and Sus scrofa (ssc). The seed region (2–8 nt) is highlighted in a gray box and underlined. (B) Predicted targets of mmu-miR-423-3p in the 3′-UTR of Cox6a2, Ndufb7, and Ndufs5 (RNAhybrid). The prediction criteria include free energy ≥ -20 kcal/mol and allowing the G:U wobble base-pair and bulging nucleotides in the seed region. The seed match is underlined.

Our microarray and correlation analysis indicated that expression of miR-423-3p decreased > 2-fold (FDR < 0.05) and was negatively correlated with the up-regulation of mitochondrial energy metabolism-related genes throughout the course of C2C12 myotube induction. *In silico* investigation of miR-423-3p showed that the miRNA is highly conserved across several species (Fig 7A) with middle abundance of the miRNA. Moreover, the target genes *Cox6a2*, *Ndufb7*, and *Ndufs5* in our energy metabolism gene list (see also Table 1) each possess at least one predicted binding site for miR-423-3p in the 3′-UTR (RNAhybrid; Fig 7B). To our knowledge, miR-423-3p has not yet been associated with ATP metabolism. Therefore, miR-423-3p was selected for functional validation.

### Overexpression of miRNA-423-3p mimic negatively regulated ATP level in C2C12 differentiation

**Fig 8:**
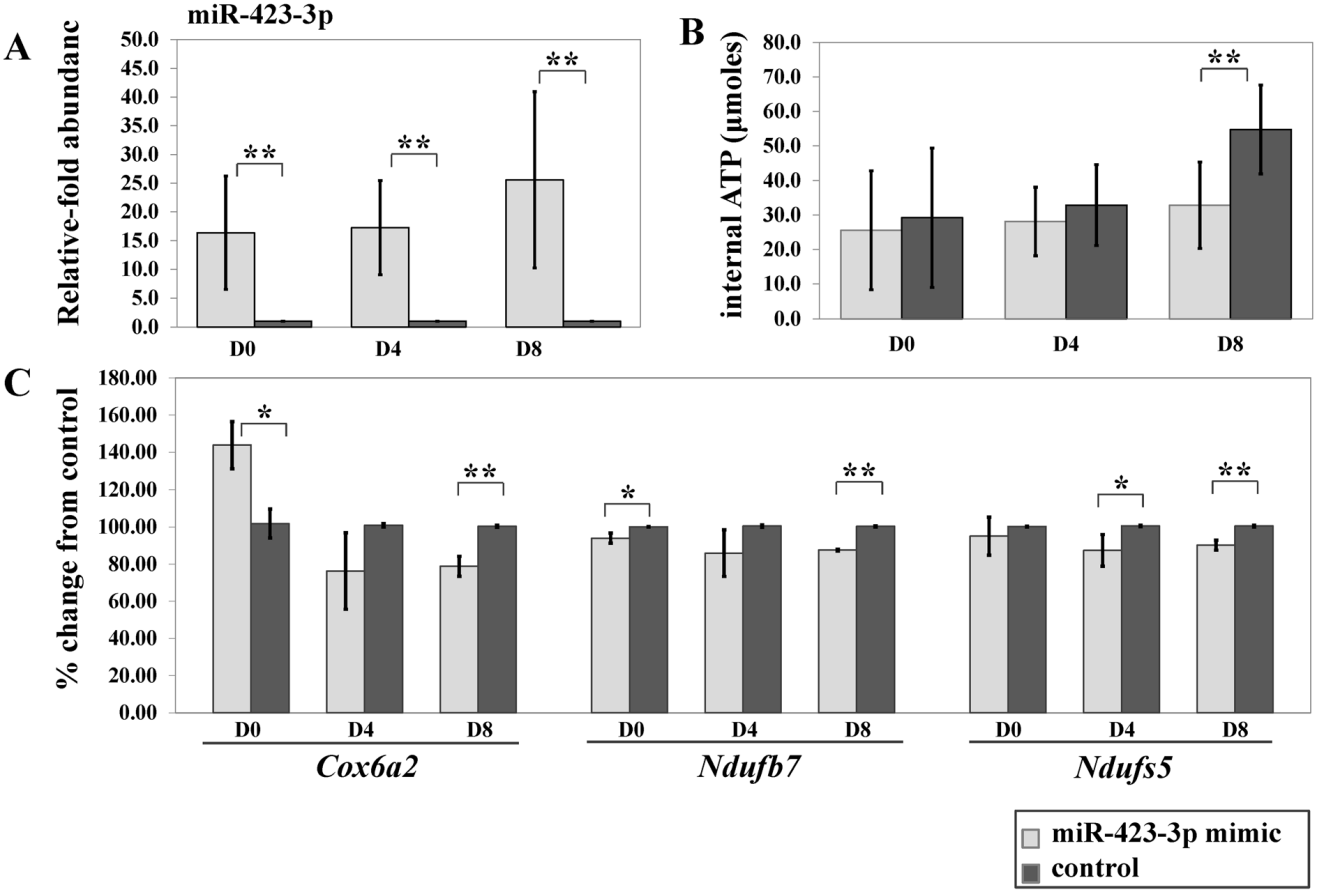
Transfection of synthetic miR-423-3p mimic negatively regulates Cox6a2, Ndufb7, and Ndufs5 as well as ATP level during C2C12 myotube induction. (A) Relative abundance of miRNA-423-3p by qPCR post miRNA-423-3p mimic transfection (n = 3). (B) 24 hours post-transfection, the cellular ATP level was lower (not significant) in the miR-423-3p mimic-transfected cells compared to the negative control at D0 and D4 and was significantly lower at D8. (C) Significant down-regulation of Cox6a2, Ndufb7, and Ndufs5 by qPCR 24 hours post-transfection. * and ** denotes p-values ≤ 0.05 and ≤ 0.01, respectively. All values are presented as mean ± SEM (n = 3).

To determine whether miR-423-3p regulates ATP metabolism, synthetic miRNA-423-3p mimics were transfected into C2C12 cells one day prior to differentiation induction and on D3 and D7 post-induction. A random-sequence double-stranded RNA oligonucleotide was used as a negative control and baseline at each time point. Gene expression analysis by real-time PCR for miR-423-3p, *Cox6a2*, *Ndufb7*, and *Ndufs5* and ATP measurements (see experimental scheme, Fig 1B) were performed 24 hours after transfection. miR-423-3p expression was detected in cells after transfection, and endogenous miR-423-3p expression was significantly lower in the negative control (Fig 8A). ATP levels tended to decrease in miR-423-3p mimic-treated cells compared to the negative control at D0 and D4 and were significantly lower at D8 (Fig 8B). Additionally, expression of *Cox6a2*, *Ndufb7*, and *Ndufs5* was significantly down-regulated at D8 (Fig 8C). All the expression of *Cox6a2*, *Ndufb7*, and *Ndufs5* in miR-423-3p mimic-treated cells experiment was in the tendency lower than control except at D0 of *Cox6a2*. The fact is that the transcript abundance of *Cox6a2* at D0 was very low with about 85 transcripts on average in the mimic experiment compared to control with about 59 transcripts. With these levels of transcripts, which was assumed as not expressed, the observed difference are biologically not relevant. The transcript abundant of *Cox6a2* at D4 and D8 ranged from 7000 to 15000 transcripts. These results suggest that miR-423-3p modulates the ATP level partly by regulating the expression of *Cox6a2*, *Ndufb7* and *Ndufs5*.

### MiR-423-3p direct target validation

Luciferase reporter assay was used to experimentally validate *Cox6a2*, *Ndufb7* and *Ndufs5* as a direct target gene for miR-423-3p (Table 2). These 3 genes were selected due to its high fold change during myogenic differentiation in C2C12 cells and hence they are likely to contribute a significant impact on ATP regulation. Moreover, two miR-423-3p binding sites spaced by only a 92 bp stretch were predicted on the 3´-UTR of *Cox6a2*. The luciferase reporter assay results indicated the binding activity of the predicted target sites and its influence on translation of the reporter gene as shown in S1 Table. This data suggests *Cox6a2* as a direct target of miR-423-3p. However, our luciferase reporter assay results could not confirm *Ndufb7* and *Ndufs5* as a direct target of miR-423-3p (data not shown) possibly influenced by background noises of COS-7 endogenous miRNAs and/or universal binding sites residing on the target sequence tested.

### Knockdown *Cox6a2* and ATP levels

**Fig 9:**
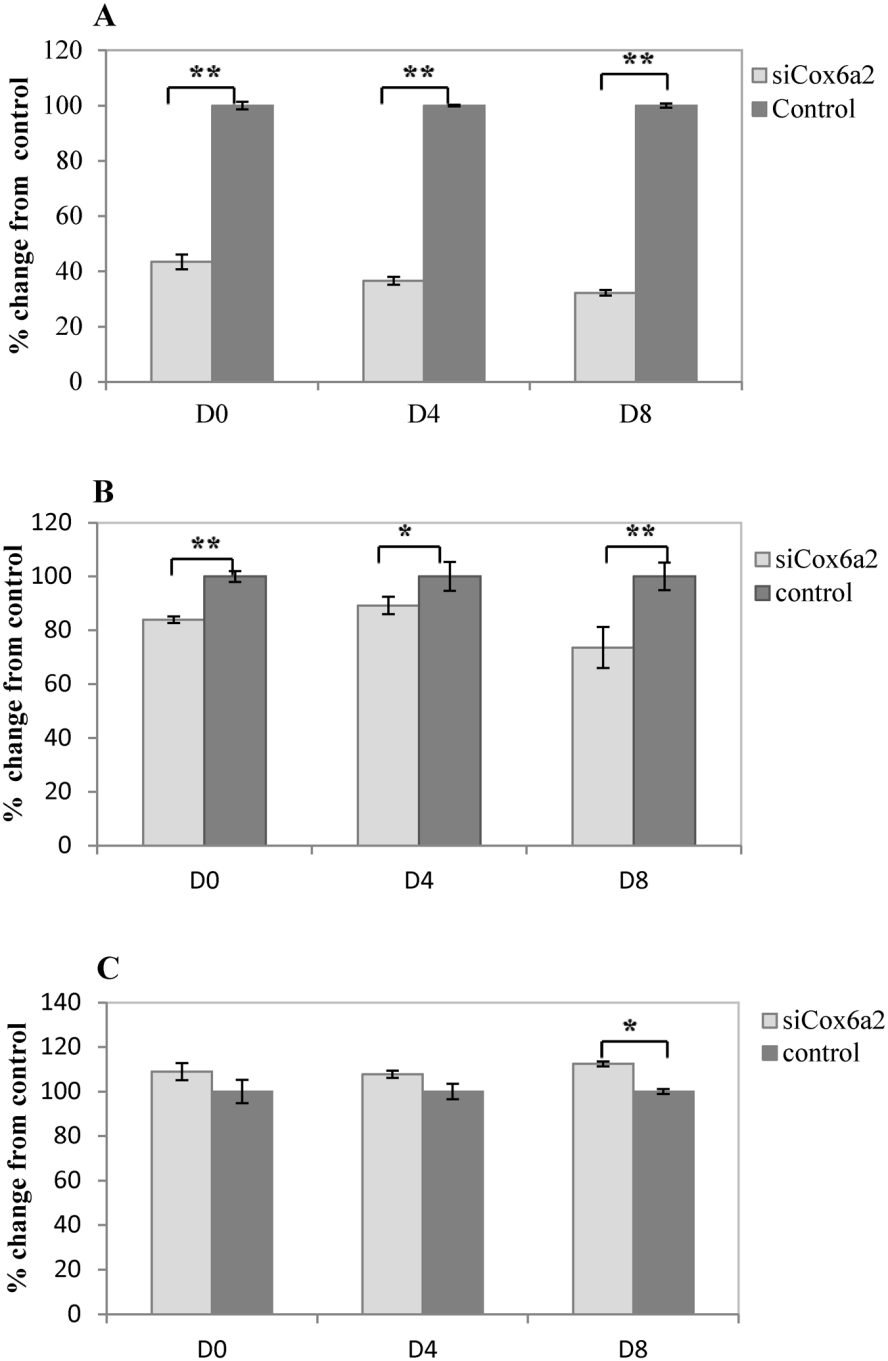
Knockdown of *Cox6a2* by RNA interference reveals regulation of ATP levels. siRNAs were designed to target *Cox6a2* and transfected into murine C2C12 muscle cells in vitro. Relative mRNA expression of Cox6a2 was measured by qPCR 48 hours after transfection. Expression was normalized to Hprt1 and Ppia internal controls. (A) Expression of *Cox6a2* was significantly reduced relative to its expression in control cells at 48 hours post-transfection of siRNA. (B) ATP levels and (C) ADP/ATP ratio were measured at D0, D4, and D8. All values are presented as mean ± SEM (n = 3).

To validate this gene, RNAi was used to knockdown *Cox6a2* expression in vitro in the C2C12 murine muscle cell line. Subsequently, relative expression of *Cox6a2* and ATP levels as well as ADP/ATP ratio were measured. siRNA targeting Cox6a2 significantly inhibited its expression to 43% (D0), 36% (D4) and 32% (D8) relative to control cells (Fig 9A). Further, Cox6a2 inhibition resulted in significantly decreased ATP levels compare to control at D0 (*p* = 0.0007), D4 (*p* = 0.027) and—most significantly—on D8 (*p* = 0.0002) (Fig 9B). ADP/ATP ratios of Cox6a2 knockdown groups were higher than control groups of D0 and D4. At D8, ADP/ATP ratios was significantly higher than controls (*p* = 0.031) (Fig 9C).

## Discussion

Using differentiating C2C12 myoblasts, we identified correlations between miRNAs, target mRNAs, and cellular ATP levels. Importantly, C2C12 myoblasts exhibited changing ATP levels during differentiation, gradually increasing at first (days 0–5), then rapidly (days 6–8) reaching 200% of the baseline level, demonstrating the increased production of ATP during induced differentiation. Previous work on ATP level and myogenic differentiation showed that extracellular ATP has an inhibitory effect on cell proliferation while simultaneously promoting myogenic differentiation of satellite cells [22]. The changing ATP level during myoblast differentiation provided a good model to search for potential regulators of ATP production.

In the correlation analyses of miRNA expression changes and ATP level, we first considered down-regulated miRNAs that were negatively correlated with the increased ATP level. Of the miRNAs identified in this group, miR-15a shares its seed sequence with that of miR-15b, which has been reported to modulate cellular ATP levels and degenerate mitochondria by targeting the down-regulation of *Arl2* in rat myocytes [23]. miR-301 and miR-17-3p function in mitochondrial metabolism by regulating ATPase and translocase [24]. Consistent with previous findings [25], the expression of miR-140, a chondrocyte-specific marker [26], decreased during myogenic differentiation. MiR-423-3p was found overexpressed in primary laryngeal carcinoma cell [27] and promotes cell growth and cell cycle in hepatocellular carcinoma cells [28]. Recently study reported that miR-423-3p was significantly correlated with the activity of caspase-3, an indicator of apoptosis in brain [29].

On the other hand, the miRNAs that were up-regulated and positively correlated with ATP level during C2C12 myogenic differentiation have not been well-described. Of these, miR-206 and miR-1 are muscle-specific miRNAs with important functions in muscle development, as regulators of cell proliferation and differentiation during myogenesis [30–32].

Because mitochondria produce ATP through oxidative phosphorylation to provide energy for cellular activities, like growth and differentiation, our microarray expression analysis focused on mitochondrial energy-metabolism genes. A number of genes were differentially expressed during C2C12 myogenic differentiation; however, only *Cox6a2*, *Ndufb7*, *Ndufs5*, *Ndufv1*, *Bcs1l*, *Ndufs4*, and *Cox4i2* were significantly up-regulated and simultaneously correlated with increased ATP throughout D0-D8.

*Cox4i2* and *Cox6a2* are nuclear genes localized in the inner mitochondrial membrane and specifically expressed in heart and muscle tissues. The COX complex functions in electron transfer in oxidative phosphorylation, which is responsible for 90% of ATP synthesis for muscle energy [33]. The COX4I2 protein encoded by *Cox4i2* is up-regulated in a limited-oxygen environment to increase ATP levels and enhance the efficiency of cellular respiration [34]. *Cox6a2*, expressed in heart and skeletal muscle, encodes one of thirteen subunits of the respiratory chain complex IV protein, the COX6A subunit. It has been implicated in stimulating enzymatic activity of the functional complex IV and it directly affects ROS production in skeletal muscle [33]. *Cox6a2* null mice have severely reduced skeletal muscle complex IV activity, decreased ATP levels, enhanced respiratory uncoupling, and increased thermogenesis, energy expenditure, mitochondrial biogenesis, and muscle-fiber type switching. These changes explain the unique phenotype of the null mice, which includes leanness and resistance to diet-induced obesity [33]. In this study knowndown of *Cox6a2* (siCox6a2) in C2C12 muscle cell culture also resulted in decreased ATP levels. Additional, ADP/ATP ratio, an indicator for apoptosis, in siCox6a2 is significant higher than in control at D8.

*Ndufb7*, *Ndufs5*, *Ndufv1*, and *Ndufs4* are members of the NADH dehydrogenase (ubiquinone) family of mitochondrial respiratory chain complex I, which represents the largest and first complex mediating electron transfer through the electron transfer chain. These enzymes transfer electrons from NADH to ubiquinone and through the respiratory chain, resulting in the generation of ATP, which is important for energy metabolism [35,36]. *Ndufs4* and *Ndufs5* are classified in the “iron—sulfur group” with a phosphorylation function. Wheras *Ndufb7* is classified in the “hydrophobic group”, *Ndufv1* is classified in the “flavoprotein group” with NADH-binding and oxidizing properties [36,37].

A correlation analysis (considering both positive and negative relationships) between the expression of miRNAs and mitochondria-related mRNAs during myogenesis was used as the first line of evidence to build a miRNA-mRNA network. A negative correlation interaction between an miRNA and an mRNA could imply a direct regulation; in contrast, a positive correlation interaction could suggest an indirect regulation via (unknown) intermediate pathways or due to neighboring position in the genome. Additionally, positive correlation interactions can indicate direct positive regulation of mRNA transcript abundance by miRNAs [21]. The finding of significant negative correlations of miR-15a and miR-16a with *Cox4i2*, *Cox6a2*, *Ndufb7*, *Ndufv1*, and *Ndufs4* is supported by previous work identifying miR-15 and miR-16 as ATP modulators affecting oxygen consumption [23] as well as direct regulators of *Bcl2* to induce apoptosis through the regulation of mitochondrial function [23,38].

## Conclusion

Our study provides a list of miRNA and mRNA which are cellular ATP regulators and which target genes involved in mitochondrial energy metabolism. Analyzing each miRNA-mRNA correlation interaction to identify mRNAs targets with 3′-UTRs containing potential binding sites for the corresponding correlated miRNAs revealed three mitochondria-related genes (*Ndufb7*, *Ndufs5*, and *Cox6a2*) sharing at least one binding site on the 3′ UTR for the same miRNA: miR-423-3p. After validation of these predicted targets (*Cox6a2*, *Ndufb7*, and *Ndufs5*), *Cox6a2* was confirmed as direct target of miR-423-3p. Additional, knowndown of *Cox6a2* resulted in decrease ATP levels and increase ADP/ATP. We therefore concluded that *Cox6a2* compose a potential miRNA-mRNA network for cellular ATP production and energy metabolism. miR-423-3p has been previously shown to involve in promoting cell growth and cell cycle progression [28]. This supports our hypothesis that down-regulation of miR-423-3p is required for C2C12 myogenic differentiation. Overexpression of miR-423-3p during C2C12 myogenic differentiation resulted in dramatically lower ATP levels and significantly lowers expression of *Cox6a2*, *Ndufb7*, and *Ndufs5*. Only *Cox6a2* could be confirmed as a direct target of miR-423-3p. All together our results suggest for the first time that miR-423-3p modulates ATP production by targeting *Cox6a2*, as an indicator of cell apoptosis.

## Supporting Information

S1 Table Luciferase reporter gene assay using the psiCHECKTM-2 vector system. (DOC)
