# Supplementary material for: MicroRNAs Regulate Cellular ATP Levels by Targeting Mitochondrial Energy Metabolism Genes during C2C12 Myoblast Differentiation
Source: PLoS One. 2015 May 26;10(5):e0127850. doi: 10.1371/journal.pone.0127850 (PMC4444189; doi:10.1371/journal.pone.0127850)
Supplement: S1 Table — (DOC) [file pone.0127850.s001.doc]

Table S1. Luciferase reporter gene assay using the psiCHECK^TM^-2 vector system.

| Co-transfection | Luciferase Activity (RLU), background subtracted | | Ratio (R/F) | Normalized fold change | t-test  (p-value) |
| --- | --- | --- | --- | --- | --- |
|  | Renilla (R) | Firefly (F) |  |  |  |
| 100 ng  psiCHECK2-Cox6a2 +  50 nM  miR-423-3p mimic | 337730  362242  361543  356738 | 749224  799790  733719  745737 | 0.451  0.453  0.493  0.478 | 0.86 | 0.002 |
| 100 ng  psiCHECK2-Cox6a2 +  50 nM  AllStars Negative control | 484303  477292  471499  456971 | 912050  891424  819462  868057 | 0.531  0.535  0.575  0.526 | 1.00 |  |
